# Involvement of Rho-associated protein kinase (ROCK) and bone morphogenetic protein-binding endothelial cell precursor-derived regulator (BMPER) in high glucose-increased alkaline phosphatase expression and activity in human coronary artery smooth muscle cells

**DOI:** 10.1186/s12933-015-0271-7

**Published:** 2015-08-12

**Authors:** Yuya Terao, Seimi Satomi-Kobayashi, Ken-ichi Hirata, Yoshiyuki Rikitake

**Affiliations:** Division of Cardiovascular Medicine, Department of Internal Medicine, Kobe University Graduate School of Medicine, 7-5-1 Kusunoki-cho, Chuo-ku, Kobe, 650-0017 Japan; Division of Signal Transduction, Department of Biochemistry and Molecular Biology, Kobe University Graduate School of Medicine, 7-5-1 Kusunoki-cho, Chuo-ku, Kobe, 650-0017 Japan

**Keywords:** ALP, BMPER, High glucose, ROCK, Statin, Vascular smooth muscle cells

## Abstract

**Background:**

Vascular calcification is an independent risk factor for cardiovascular disease. Diabetes mellitus increases the incidence of vascular calcification; however, detailed molecular mechanisms of vascular calcification in diabetes mellitus remain unknown. We recently reported that bone morphogenetic protein-binding endothelial cell precursor-derived regulator (BMPER) regulates osteoblast-like *trans*-differentiation of human coronary artery smooth muscle cells (HCASMCs).

**Methods:**

We investigated the effect of a hydroxymethylglutaryl-coenzyme A reductase inhibitor (statin), commonly used in patients with atherosclerotic diseases and diabetes mellitus, on alkaline phosphatase (ALP) mRNA expression in aortas of streptozotocin-induced diabetic mice. We also investigated the effects of the statin, Rho-associated protein kinase (ROCK) inhibitors and BMPER knockdown on ALP mRNA expression and activity in HCASMCs cultured in high glucose-containing media.

**Results:**

Alkaline phosphatase mRNA expression was increased in aortas of streptozotocin-induced diabetic mice, and the increase was inhibited by rosuvastatin. ALP mRNA expression and activity were increased in HCASMCs cultured in high glucose-containing media, and the increases were suppressed by rosuvastatin. This suppression was reversed by the addition of mevalonate or geranylgeranyl pyrophosphate, but not farnesyl pyrophosphate. High glucose-increased ALP mRNA expression and activity were suppressed by ROCK inhibitors. Moreover, BMPER mRNA expression was increased in diabetic mouse aortas and in HCASMCs cultured in high glucose-containing media, but was not inhibited by rosuvastatin or ROCK inhibitors. Knockdown of BMPER suppressed high glucose-increased ALP activity, but not ROCK activity in HCASMCs.

**Conclusions:**

There are at least two independent pathways in high glucose-induced ALP activation in HCASMCs: the Rho–ROCK signaling pathway and the BMPER-dependent pathway.

## Background

Vascular calcification is one of the common pathological changes of atherosclerosis and is an independent risk factor for cardiovascular disease. The incidence of vascular calcification increases with aging, smoking, hemodialysis, and diabetes mellitus. However, detailed molecular mechanisms of vascular calcification in diabetes mellitus remain poorly understood. In diabetes mellitus, high glucose promotes *trans*-differentiation of vascular smooth muscle cells, which is thought to cause vascular calcification, and hyperinsulinemia and elevated advanced glycosylation end products may play a role in vascular calcification [1].

Hydroxymethylglutaryl-coenzyme A (HMG-CoA) reductase inhibitors, statins, are drugs that lower cholesterol levels by inhibiting HMG-CoA reductase. Statins are commonly used in patients with atherosclerotic diseases and diabetes mellitus. It is widely recognized that statins have pleiotropic effects unrelated to cholesterol-lowering effects, such as anti-inflammatory and anti-oxidative effects [2]. In addition to inhibiting cholesterol synthesis, statins also block the synthesis of isoprenoid intermediates such as farnesyl pyrophosphate (FPP) and geranylgeranyl pyrophosphate (GGPP) [3]. FPP and GGPP serve as important lipid attachments for the posttranslational modification of a variety of proteins, including small GTPases [4]. Modification with FPP is necessary for proper localization of Ras family proteins, whereas GGPP is required for Rho, Rab, and Rap family proteins. In vitro studies have demonstrated that statins inhibit Rho by inhibiting GGPP, and thereby suppress its effector Rho-associated protein kinase (ROCK) [5].

It was reported that statins inhibit in vitro calcification of vascular smooth muscle cells induced by inorganic phosphate [6], inflammatory mediators [7], vitamin D_3_ and warfarin [8], and transforming growth factor (TGF)-β [9]. However, with regard to the inhibitory effects of statins on vascular calcification, conflicting results have been reported between in vitro research and clinical studies. Several clinical studies using electron beam tomography have shown that statins reduce coronary artery calcification [10–12]. However, three subsequent randomized trials using electron beam tomography have shown no inhibitory effects of statins on coronary artery calcification [13–15]. Furthermore, a recent analysis of eight randomized trials using intravascular ultrasonography showed that statins promoted coronary artery calcification [16].

Bone morphogenetic protein (BMP)-binding endothelial cell precursor-derived regulator (BMPER) is a secretory protein that is known to bind to BMP-2, 4 and 6 [17]. BMPER can work as either an activator or an inhibitor of BMP signaling, depending on its concentrations and environment [18]. We have reported that BMPER is a regulator of the osteoblast-like *trans*-differentiation of human coronary artery smooth muscle cells (HCASMCs) [19]. Knockdown of BMPER inhibits, whereas addition of BMPER enhances the osteoblast-like *trans*-differentiation of HCASMCs.

Here, we investigated the effects of a statin, ROCK inhibitors and BMPER knockdown on alkaline phosphatase (ALP) mRNA expression and activity in HCASMCs cultured in high glucose-containing media to examine the potentially critical roles of the Rho–ROCK signaling pathway and BMPER in vascular calcification in diabetes mellitus.

## Methods

### Mice

All animal experiments were approved by the Institutional Animal Care and Use Committee and carried out according to the Kobe University Animal Experimental Regulations (P140605). Streptozotocin (STZ)-induced type I diabetic mice were produced as described previously [20] with slight modification. Briefly, male C57BL/6J mice (10-week-old) were intraperitoneally injected with STZ in distilled water (200 mg/kg; Wako, Osaka, Japan) or the equal volume of vehicle as a control. Blood samples were taken from mouse lateral tail vein and blood glucose was measured by OneTouch Ultra glucometer (LifeScan, Wayne, PA, USA). Fasting blood glucose was measured at 4 and 8 days after the first STZ injection, and additional STZ injections (250 mg/kg) were given when the fasting blood glucose was less than 250 mg/dl. Rosuvastatin, kindly provided by AstraZeneca, was dissolved in drinking water, which was available ad libitum, and administered with 7.2 mg/kg body weight/day for 14 days. After being anesthetized intraperitoneally with 2.5 % 2,2,2-tribromoethanol (1.6 ml/100 g), the mice were transcardially perfused with physiological saline solution and the aortas were isolated.

### Cell culture

Human coronary artery smooth muscle cells (Lonza, Basel, Switzerland) were cultured at 37 °C in Dulbecco’s modified Eagle’s medium (Nacalai, Kyoto, Japan) (glucose; 5.5 mM) supplemented with 15 % fetal bovine serum (Life Technologies, Carlsbad, CA, USA), 100 IU/ml penicillin and 100 g/ml streptomycin (Nacalai) (normal glucose-containing media). High glucose-containing media (final 25 mM) was made by addition of 19.5 mM glucose to normal glucose-containing media. In like manner, 19.5 mM mannitol (Sigma-Aldrich, St. Louis, MO, USA) was added to normal glucose-containing media as an osmolality control (mannitol-containing media). Cells between passages 8 and 14 were used for the experiments. Unless otherwise noted, cells were cultured for the indicated time periods without changing media. In some experiments, HCASMCs were cultured with rosuvastatin (10 µM), mevalonate (Sigma-Aldrich) (100 µM), FPP (Sigma-Aldrich) (10 µM), GGPP (Sigma-Aldrich) (10 µM), fasudil (Wako) (10 µM) and Y-27632 (Wako) (10 µM). Human umbilical vein endothelial cells (HUVECs) (Lonza) were cultured at 37 °C in the EGM-2 BulletKit (Lonza).

### Small interfering RNA (siRNA) experiments

Knockdown of BMPER by siRNAs was performed as described previously [19]. HCASMCs were transfected with Stealth siRNAs for BMPER (Life Technologies) using Lipofectamine RNAiMAX (Life Technologies) according to the manufacturer’s instructions. A Stealth siRNA non-silencing negative control (Life Technologies) was used as a control. Forty-eight hours after transfection, HCASMCs were subjected to each experiment.

### Real-time polymerase chain reaction (PCR)

Real-time PCR was performed as described previously [19]. Total mRNAs were extracted from mouse aortas and HCASMCs using TRIzol Reagent (Life Technologies) and subjected to real-time PCR using a 7500 Real-Time PCR System (Life Technologies) with a SYBR Premix Ex Taq II (Tli RNaseH Plus) (Takara Bio, Otsu, Japan). The following primers were used: mouse ALP: forward, 5-ACACCTTGACTGTGGTTACTGCTGA-3 and reverse, 5-CCTTGTAGCCAGGCCCGTTA-3; mouse BMPER: forward, 5-ATTACCTGCTGCGTCTTGCT-3 and reverse, 5-TTCTCTCACGCACTGTGTCC-3; mouse GAPDH: forward, 5-GACCCCTTCATTGACCTCAACTAC-3 and reverse, 5-TTTCTTACTCCTTGGAGGCCATGT-3; and human ALP: forward, 5-GGACCATTCCCACGTCTTCAC-3 and reverse, 5-CCTTGTAGCCAGGCCCATTG-3; human BMPER: forward, 5-AGGACAGTGCTGCCCCAAATG-3 and reverse, 5-TACTGACACGTCCCCTGAAAG-3; human glyceraldehyde-3-phosphate dehydrogenase (GAPDH): forward, 5-CTGATGCCCCCATGTTCGTC-3 and reverse, 5-CACCCTGTTGCTGTAGCCAAATTC-3. Primer pairs were purchased from Takara Bio. GAPDH was used for standardization, and the comparative threshold method was used to assess the relative abundance of the targets.

### ALP staining

Alkaline phosphatase staining was performed essentially as described previously [19]. HCASMCs cultured in 24-well plates were washed with phosphate-buffered saline, and fixed in 4 % paraformaldehyde for 2 min. The cells were incubated in substrate working solution [100 mg/ml naphthol AS-MX phosphatase (Sigma-Aldrich), 600 mg/ml fast red TR salt (Sigma-Aldrich), 0.5 % (v/v) *N*,*N*-dimethylformamide, 2 mM MgCl_2_ and 0.1 M Tris–HCl pH 8.8] at 37 °C for 20 min. The cells were washed until the intense red color became indicative. The ratio of ALP-positive area was calculated from 10 wells, with at least 500 cells counted per well, using ImageJ software.

### Western blotting

Western blotting was performed as described previously [21]. ROCK activity was measured by myosin phosphatase target subunit 1 (MYPT1) phosphorylation as described previously [22]. Rabbit anti-phospho-MYPT1 polyclonal antibody (#4563, Cell Signaling Technology, Danvers, MA, USA), rabbit anti-MYPT1 polyclonal antibody (#2634, Cell Signaling Technology) and anti-rabbit immunoglobulin G, horseradish peroxidase-linked whole antibody donkey (GE Healthcare Bioscience, Pittsburgh, PA, USA) were used at 1:1,000. The signals were detected using the Amersham Imager 600 (GE Healthcare Bioscience, Little Chalfont, UK). Densitometric analysis was performed using ImageJ software.

### Statistical analysis

All experiments were performed at least three times, and the results are expressed as mean ± standard error of the mean (SEM). Student’s *t*-test was used when comparing differences between two groups. Differences between more than three groups were analyzed by one-way analysis of variance, followed by Tukey’s or Dunnett’s multiple comparison tests, as appropriate. Values of *P* < 0.05 were considered significant.

## Results

### Rosuvastatin decreased ALP mRNA expression in aortas of STZ-induced type I diabetic mice

**Fig. 1:**
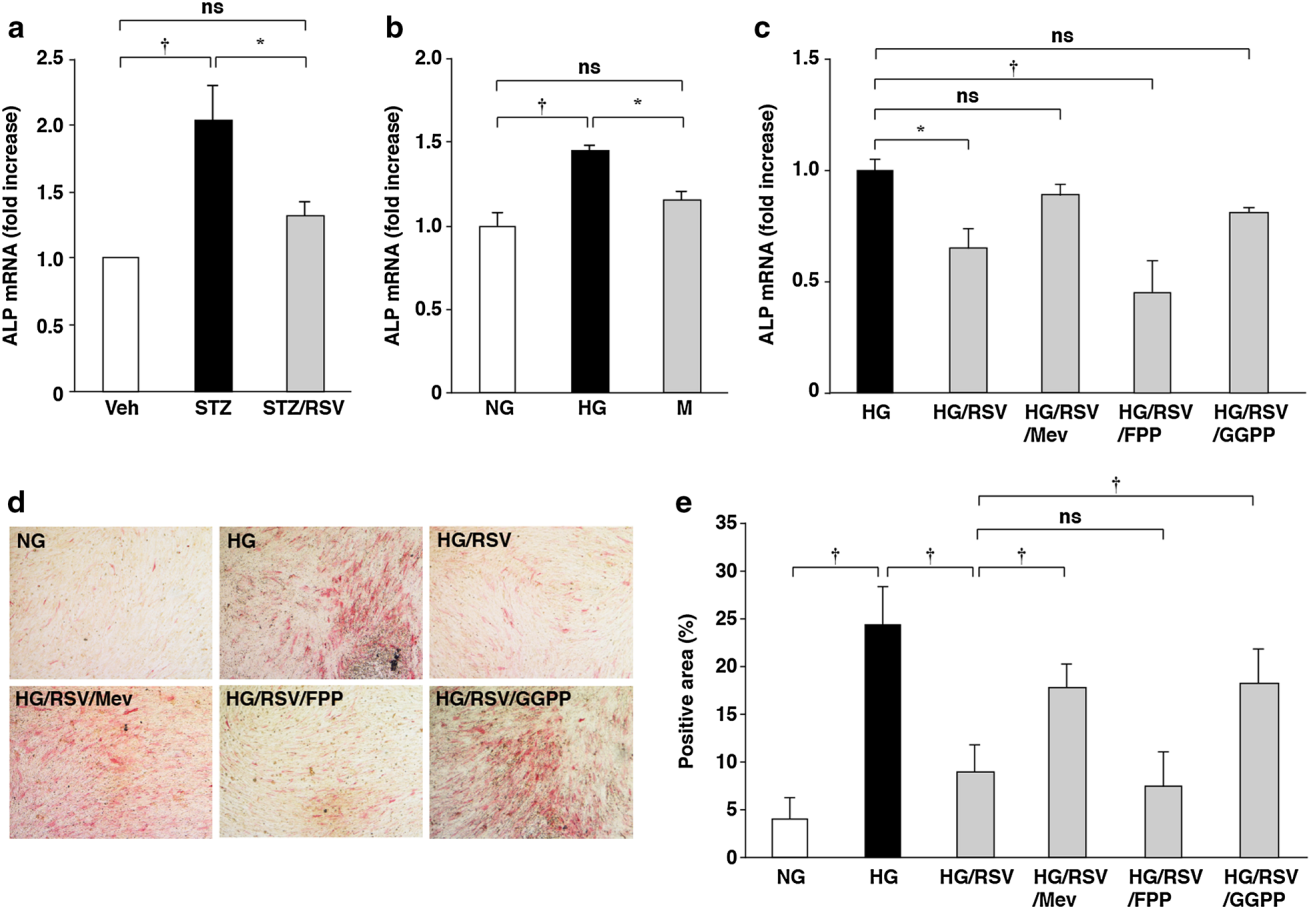
Effects of rosuvastatin, mevalonate and GGPP on high glucose-increased ALP mRNA expression and activity. **a** ALP mRNA expression in aortas of STZ-induced type I diabetic mice. The values represent the mean ± SEM (*n* = 8–11). **b** ALP mRNA expression in HCASMCs cultured for 10 days. The values represent the mean ± SEM (*n* = 3–4). **c** The inhibitory effects of rosuvastatin on high glucose-induced ALP mRNA expression, and reversal by mevalonate and GGPP. HCASMCs were cultured with rosuvastatin, mevalonate, FPP and GGPP for 5 days in high glucose-containing media. The values represent the mean ± SEM (*n* = 6). **d** The inhibitory effects of rosuvastatin on high glucose-induced ALP activity, and reversal by mevalonate and GGPP. HCASMCs were cultured for 10 days. Representative images are shown. **e** Percentages of the ALP-positive area relative to the total surface area of HCASMCs. The data are presented as mean ± SEM (*n* = 5). *Veh* vehcle, *RSV* rosuvastatin, *NG* normal glucose, *HG* high glucose, *M* mannitol, *Mev* mevalonate. *P < 0.05; ^†^ *P* < 0.01; *ns* not significant.

We investigated the effect of rosuvastatin on ALP expression in aortas of STZ-induced type I diabetic mice. Compared with control mice, ALP mRNA expression in aortas of STZ-induced type I diabetic mice given rosuvastatin was significantly increased (~2.0-fold; *P* < 0.01). Administration of rosuvastatin significantly inhibited the increase in ALP mRNA expression (*P* < 0.05) (Fig. 1a).

### Rosuvastatin suppressed high glucose-increased ALP mRNA expression and activity in HCASMCs

To elucidate molecular mechanisms of the inhibition of ALP mRNA expression in diabetic mice by rosuvastatin, we performed in vitro experiments using cultured HCASMCs. When HCASMCs were cultured in high glucose-containing media, ALP mRNA expression and activity were significantly increased and these increases were significantly suppressed by rosuvastatin (Fig. 1b, d). Because statins show pleiotropic effects by inhibiting isoprenoid synthesis, the effects of mevalonate, FPP and GGPP on the rosuvastatin-suppressed ALP expression were examined. The suppression of ALP expression by rosuvastatin was reversed by the addition of mevalonate and GGPP, but not by the addition of FPP (Fig. 1c–e). These results indicate that rosuvastatin suppresses high glucose-increased ALP mRNA expression and activity in HCASMCs, and that the effects of rosuvastatin are likely due to the inhibition of GGPP synthesis.

### ROCK inhibitors suppressed high glucose-increased ALP mRNA expression and activity in HCASMCs

**Fig. 2:**
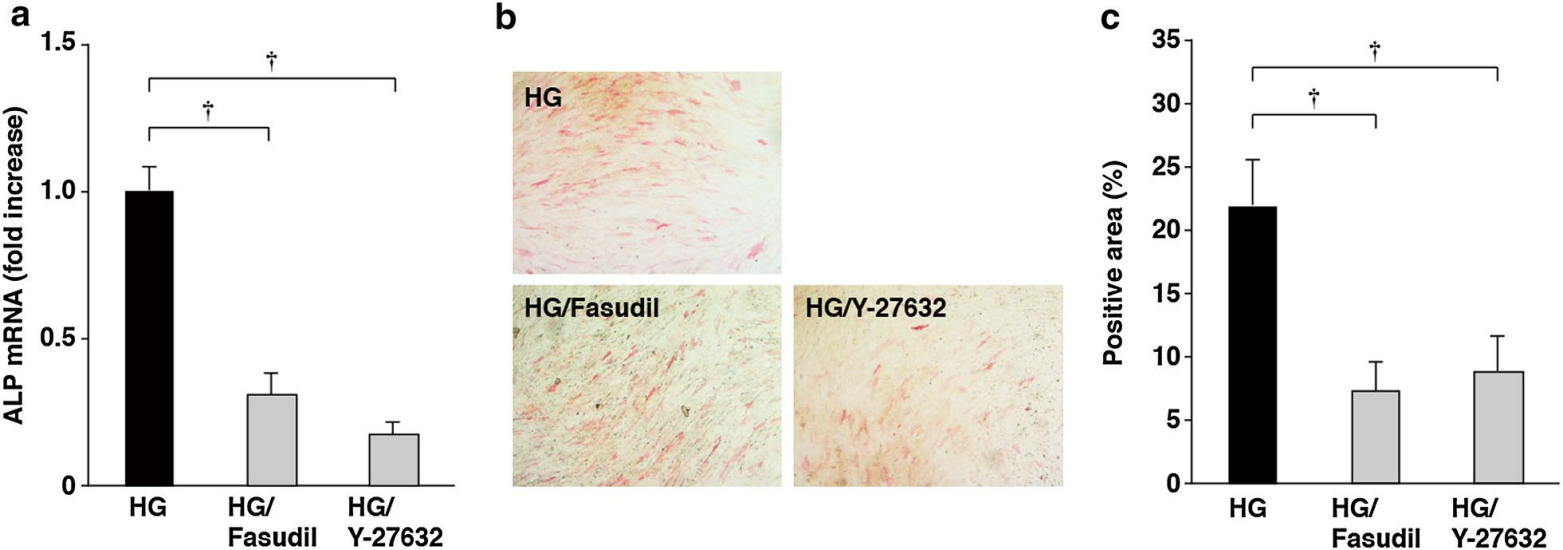
Inhibition of high glucose-increased ALP mRNA expression and activity by ROCK inhibitors. **a** Inhibition of high glucose-induced increases in ALP mRNA levels in HCSMCs by fasudil and Y-27632. HCASMCs were cultured in high glucose-containing media for 5 days. The values represent the mean ± SEM (*n* = 3). **b**, **c** Inhibition of high glucose-induced increases in ALP activity of HCSMCs by fasudil and Y-27632. HCASMCs were cultured for 10 days. Representative images of the ALP-staining (**b**) and the percentages of the ALP-positive area relative to the total surface area of HCASMCs (**c**) are shown. The data are presented as mean ± SEM (*n* = 5). ^†^ *P* < 0.01.

GGPP is required for geranylgeranylation of small G proteins such as Rho, Rac and Cdc42 [4]. In particular, inhibition of Rho and its downstream target, ROCK, has emerged as the principle mechanism underlying the pleiotropic effects of statins [22, 23]. We therefore focused on the role of the Rho–ROCK signaling pathway. To reveal whether ROCK is involved in high glucose-increased ALP expression and activity, the effects of specific ROCK inhibitors, fasudil and Y-27632, were examined. The increases in ALP mRNA expression and activity by cultivation in high glucose-containing media were effectively suppressed by the ROCK inhibitors fasudil and Y-27632 (Fig. 2a–c).

### BMPER was involved in high glucose-increased ALP activity in HCASMCs

**Fig. 3:**
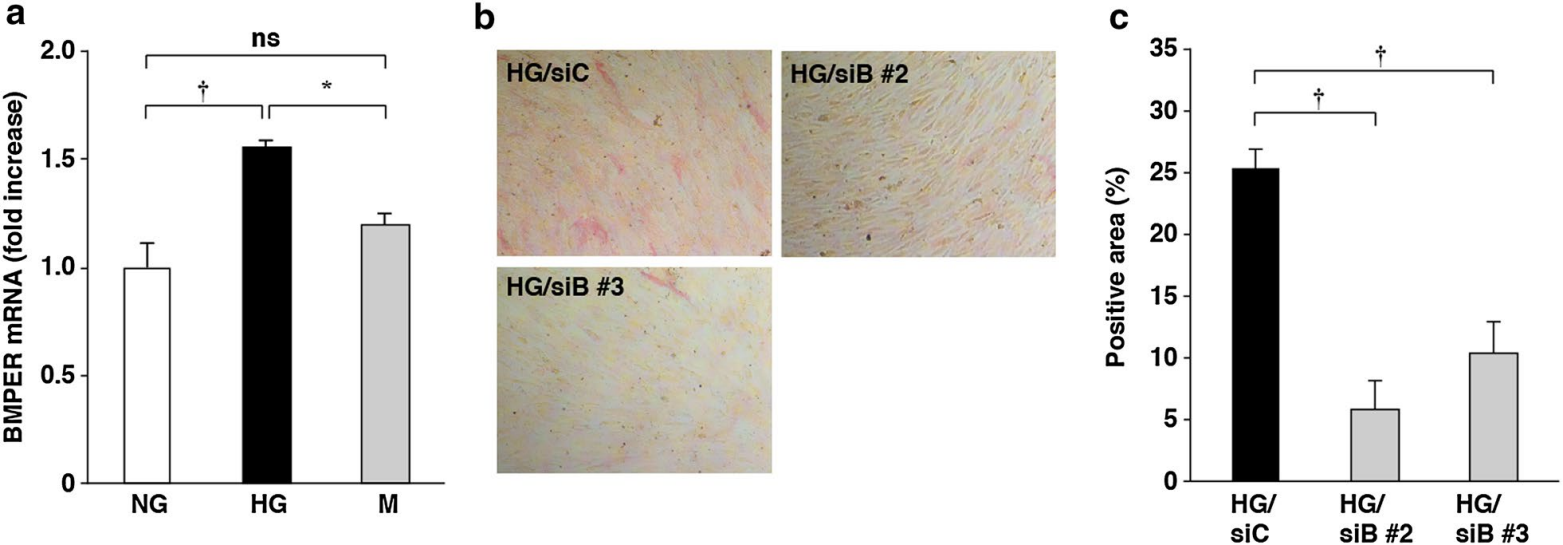
Involvement of BMPER in high glucose-increased ALP activity. **a** High glucose-induced increases in BMPER mRNA levels in HCSMCs cultured for 10 days. The values represent the mean ± SEM (*n* = 3–4). **b**, **c** Inhibition of high glucose-induced increases in ALP mRNA levels in HCSMCs by BMPER knockdown. HCASMCs cultured in high glucose-containing media for 10 days. Representative images of the ALP-staining (**b**) and the percentages of the ALP-positive area relative to the total surface area of HCASMCs (**c**) are shown. The data are presented as mean ± SEM (*n* = 4–5). *siC* control siRNA, *siB* BMPER siRNA. *P < 0.05; ^†^ *P* < 0.01; *ns* not significant.

**Fig. 4:**
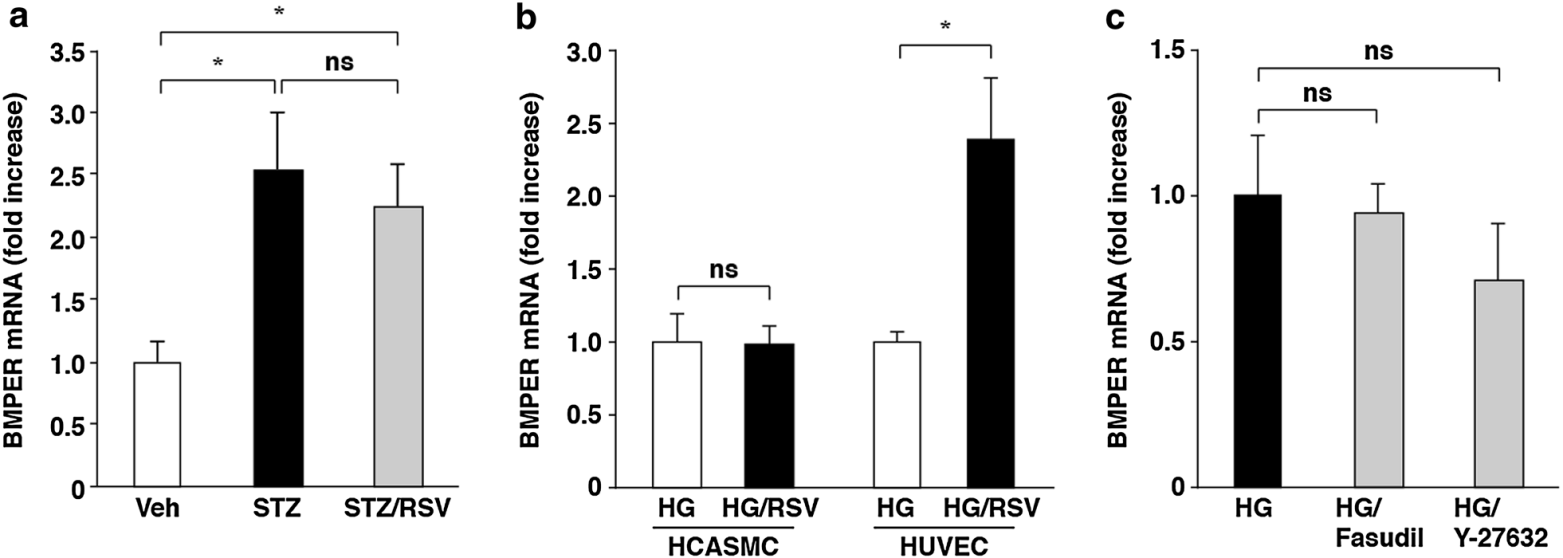
Effects of rosuvastatin and ROCK inhibitors on BMPER mRNA levels. **a** Rosuvastatin did not inhibit increases in BMPER mRNA expression in aortas of STZ-induced diabetic mice. The values represent the mean ± SEM (*n* = 9–11). **b** Rosuvastatin did not change BMPER mRNA levels in HCASMCs. HCASMCs and HUVECs were cultured with rosuvastatin in high glucose-containing media for 2 days. The values represent the mean ± SEM (*n* = 4). **c** Fasudil and Y-27632 did not change BMPER mRNA levels in HCASMCs cultured in high glucose-containing media for 5 days. The values represent the mean ± SEM (*n* = 3). *P < 0.05; *ns* not significant.

We then examined whether BMPER was involved in high glucose-increased ALP activity. BMPER mRNA levels were increased by cultivation in high glucose-containing media, but not by mannitol (Fig. 3a). The high glucose-increased ALP activity was suppressed by knockdown of BMPER by siRNAs (Fig. 3b, c). Furthermore, BMPER mRNA levels were increased in aortas of STZ-induced type I diabetic mice by 2.5-fold (*P* < 0.05) (Fig. 4a). Collectively, these results show that BMPER mRNA expression is increased by cultivation in high glucose-containing media and in diabetic mice, and indicate the involvement of BMPER in high glucose-increased ALP activity in HCASMCs.

### BMPER-mediated ALP activation was independent of the Rho–ROCK signaling pathway

To clarify the relationship between the Rho–ROCK signaling pathway and BMPER in high glucose-increased ALP activity in HCASMCs, we first examined the effect of rosuvastatin on BMPER mRNA expression. BMPER mRNA expression was not significantly inhibited by rosuvastatin in mouse aortas (Fig. 4a). BMPER mRNA expression was not changed by rosuvastatin in HCASMCs, but was significantly increased in HUVECs (Fig. 4b). The increases in BMPER mRNA expression in HUVECs were consistent with the previous report [24]. Then, the effects of ROCK inhibitors on BMPER mRNA expression were examined. ROCK inhibitors did not inhibit the high glucose-increased BMPER mRNA expression (Fig. 4c). Collectively, these results indicate that the Rho–ROCK signaling pathway is not located upstream of the high glucose-increased BMPER mRNA expression.

**Fig. 5:**
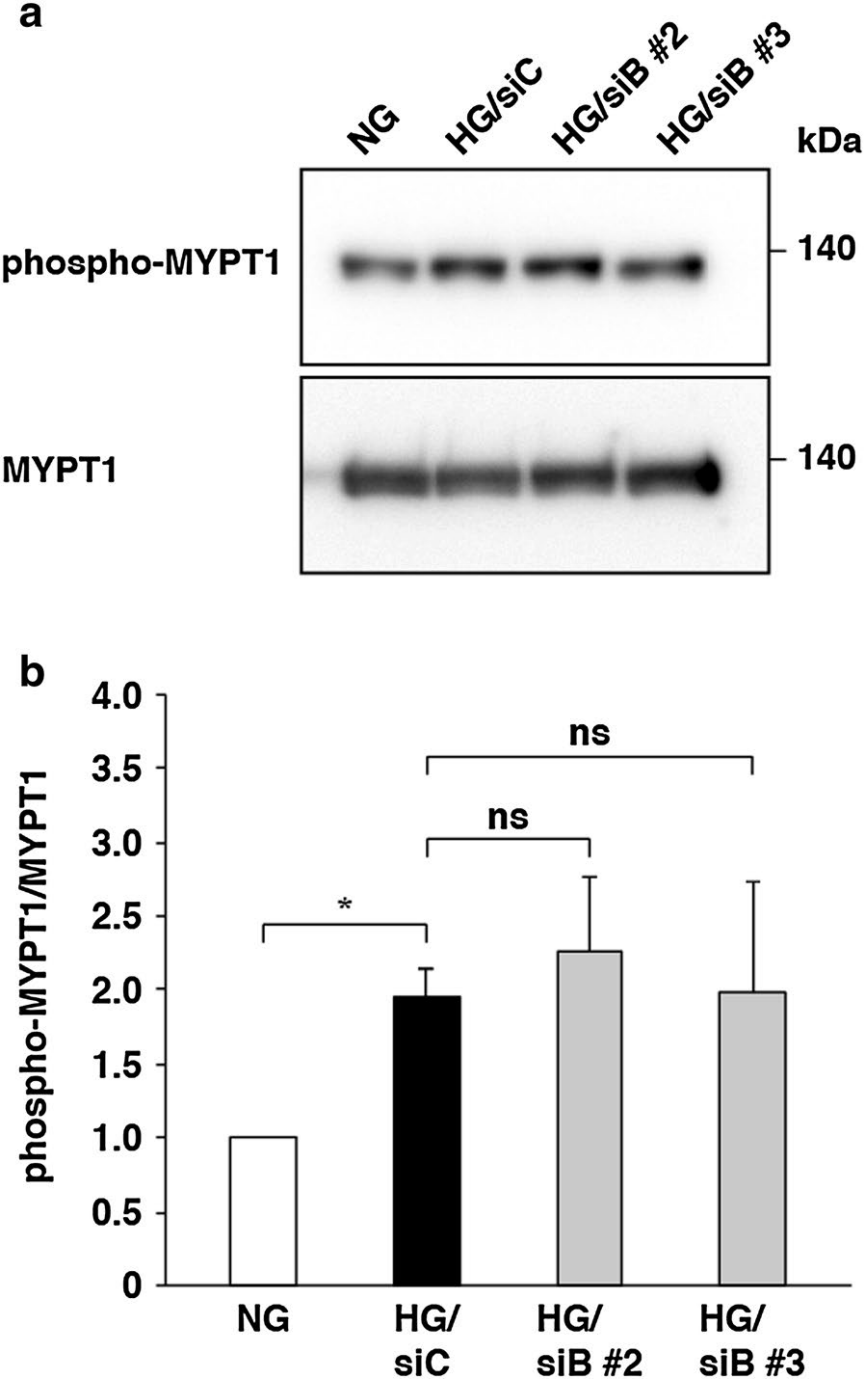
Effect of BMPER knockdown on ROCK activity. HCASMCs were cultured in high glucose-containing media for 10 days and MYPT1 phosphorylation was examined. Representative results (**a**) and densitometry (**b**) are shown. The values represent the mean ± SEM (*n* = 3). *P < 0.05; *ns* not significant.

Next, to reveal whether high glucose induces activation of the Rho–ROCK signaling pathway via BMPER, we examined MYPT1 phosphorylation. High glucose increased MYPT1 phosphorylation, but knockdown of BMPER did not inhibit MYPT1 phosphorylation (Fig. 5). Collectively, these results indicate that, although the Rho–ROCK signaling pathway is involved in high glucose-induced ALP activation in HCASMCs, BMPER-mediated signaling is another pathway independent of the Rho–ROCK signaling pathway.

**Fig. 6:**
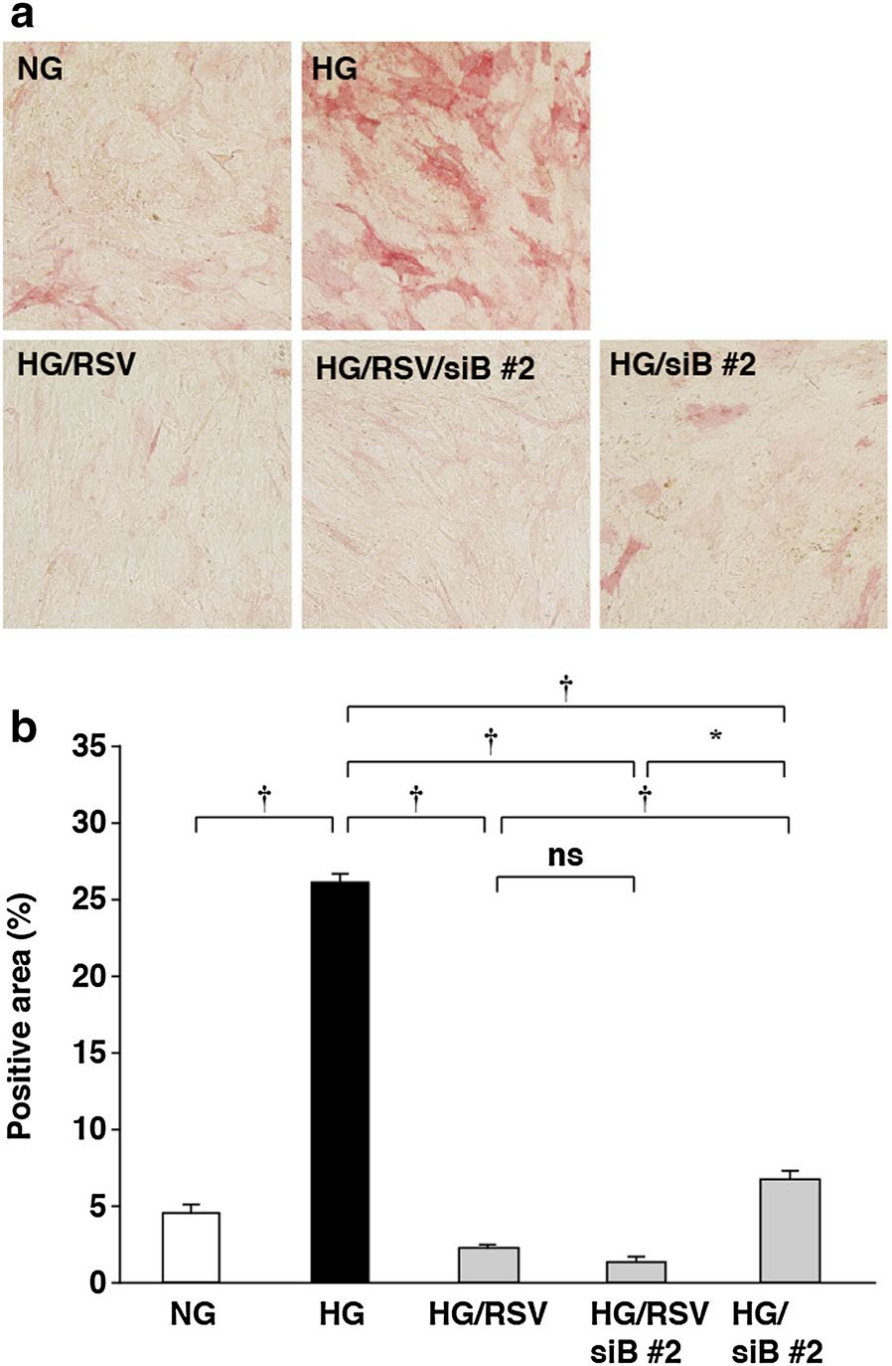
Comparison of the inhibitory effects of BMPER knockdown with rosuvastatin on high glucose-increased ALP activity. ALP activity of HCASMCs cultured with rosuvastatin, BMPER knockdown-HCASMCs, and BMPER knockdown-HCASMCs cultured with rosuvastatin was compared at day 10. Representative images of the ALP-staining (**a**) and percentages of the ALP-positive area relative to the total surface area of HCASMCs (**b**) are shown. The data are presented as mean ± SEM (*n* = 4). *P < 0.05; ^†^ *P* < 0.01; *ns* not significant.

Finally, the inhibitory effects of BMPER knockdown and rosuvastatin on ALP activity were compared. Although both BMPER knockdown and rosuvastatin showed a significant inhibition of high glucose-increased ALP activity, there still existed a significant inhibitory effect of rosuvastatin (Fig. 6a, b). Collectively, these results suggest that there were at least two pathways in high glucose-increased ALP activity: the Rho–ROCK-dependent pathway and the BMPER-dependent pathway.

## Discussion

### Critical role of the Rho–ROCK signaling pathway in high glucose-induced ALP activation

Previous reports have shown high glucose induces osteogenic changes in vascular smooth muscle cells [25–27]. Moreover, it has been reported that statins show inhibitory effects on TGF-β-induced [9], vitamin D3 and warfarin combination therapy-induced [8], and inorganic phosphate-induced [6] *trans*-differentiation of cultured vascular smooth muscle cells, which is considered to reflect vascular calcification. However, as far as we know, whether statins inhibit high glucose-induced ALP activation has not been reported. Furthermore, we show here, for the first time, the increases in BMPER mRNA expression in vascular smooth muscle cells cultured in high glucose-containing media, and BMPER involvement in high glucose-induced ALP activation, independent of the Rho–ROCK signaling pathway. GGPP is synthesized from the combination of FPP and isopentenyl pyrophosphate (IPP) and because statins block the pathway upstream of these moieties, GGPP cannot be synthesized by treatment with statins even upon FPP supplementation, as IPP is not available [3]. The restoration of rosuvastatin-inhibited ALP activation in HCASMCs by GGPP, but not by FPP, suggests the involvement of GGPP-dependent proteins such as Rho, Rac and Cdc42, rather than farnesylation-dependent proteins such as Ras. In line with this, ROCK inhibitors showed similar inhibitory effects on high glucose-induced ALP activation in HCASMCs. Thus, the Rho–ROCK signaling pathway is critical for high glucose-induced ALP activation.

In contrast to the results of the present study, Trion et al. showed a dose-dependent stimulatory effect of statins on calcification of vascular smooth muscle cells [28]. However, because in this study a 500 times higher concentration of atorvastatin was used than in Son et al. [6], who reported a protective effect of atorvastatin, calcification induced by the statin in the study done by Trion et al. may have resulted from apoptosis of vascular smooth muscle cells. In contrast to lipophilic statins like atorvastatin, hydrophilic statins such as pravastatin and rosuvastatin are reported to suppress apoptosis of various cells [29]. Pravastatin suppresses apoptosis of vascular smooth muscle cells [30–32], while rosuvastatin suppresses apoptosis of vascular endothelial cells [33], cardiomyocytes [34], and podocytes [35]. In the present study, we used rosuvastatin at the commonly-used concentration (10 µM), at which apoptosis does not occur, and therefore the rosuvastatin-induced suppression of the high glucose-increased ALP mRNA expression and activity was not due to apoptosis of HCASMCs.

### Contribution of the Rho–ROCK signaling pathway and BMPER to high glucose-induced ALP activation

**Fig. 7:**
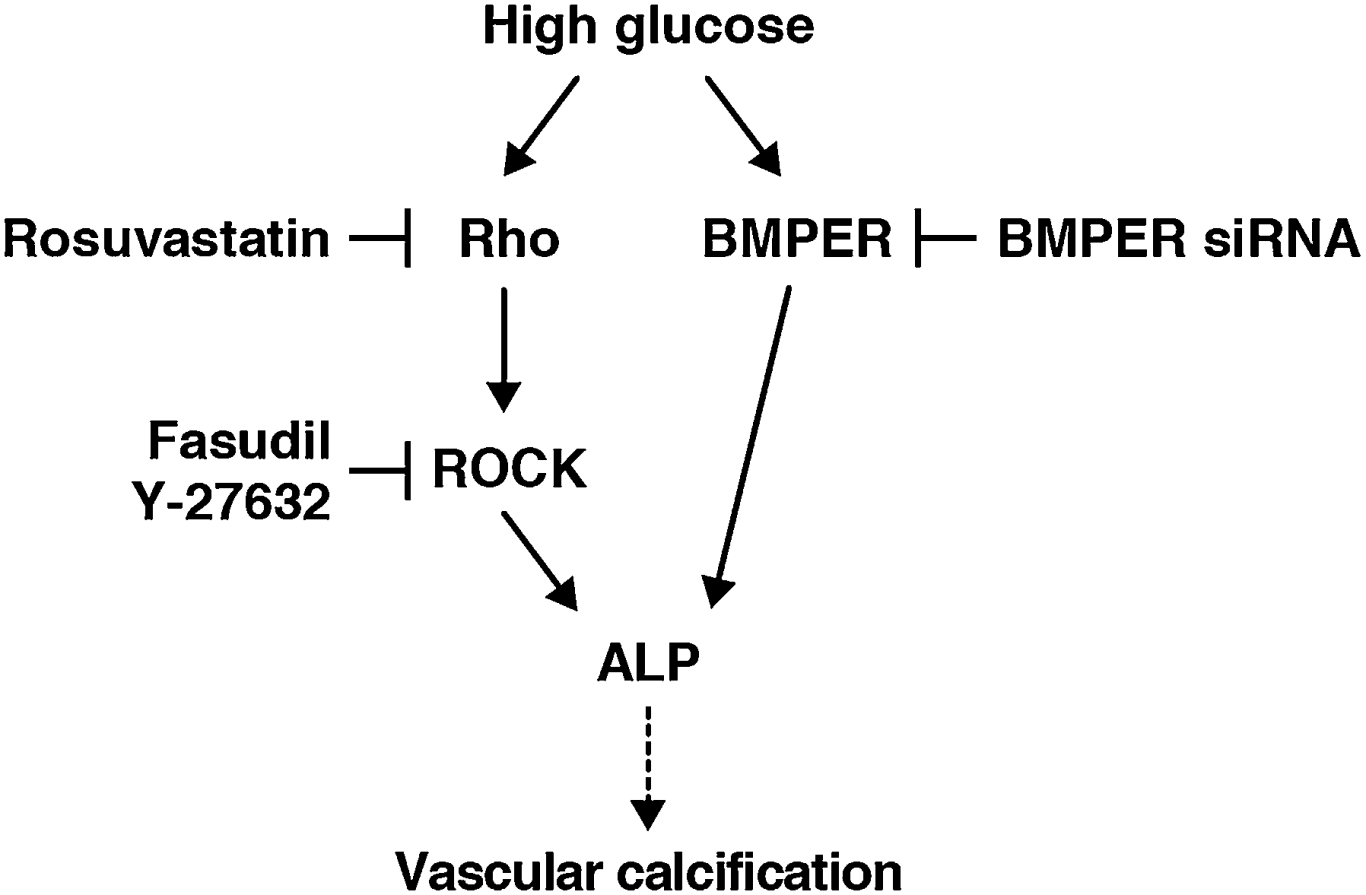
Contribution of the Rho–ROCK signaling pathway and BMPER to high glucose-induced ALP activation in HCASMCs. There are at least two independent pathways in high glucose-induced ALP activation in HCASMCs: the Rho–ROCK signaling pathway and the BMPER-dependent pathway.

Tonic ROCK mediated vasoconstriction contributes to coronary vasomotor tone in early diabetes [36], and diabetes-induced vascular dysfunction can arise via inhibition of endothelial nitric oxide synthase caused directly or indirectly due to an up-regulation of ROCK by hyperglycemia [37]. Moreover, ROCK is substantially involved in the pathogenesis of coronary vasospasm, angina pectoris, hypertension, pulmonary hypertension, and heart failure [38]. Kawamura et al. have reported that high glucose-increased osteopontin expression was mediated via the Rho–ROCK signaling pathway in vascular smooth muscle cells [25]. We hypothesized that BMPER might be regulated by the Rho–ROCK signaling pathway. However, ROCK inhibitors did not change BMPER mRNA expression. Moreover, BMPER knockdown did not change MYPT1 phosphorylation. Collectively, these results indicate that there are at least two independent pathways in high glucose-induced ALP activation: the Rho–ROCK signaling pathway and the BMPER-dependent pathway (Fig. 7). From our results we hypothesize that there may a possible new therapeutic strategy against vascular calcification, in which BMPER is inhibited. In addition, we have shown here that rosuvastatin and ROCK inhibitors did not have any effects on BMPER mRNA expression in HCASMCs. In contrast, it was reported that statins increased BMPER mRNA expression via the Rho–ROCK signaling pathway in HUVECs [24]. These conflicting results suggest that *Bmper* gene transcription might be differentially regulated between HCASMCs and HUVECs.

### Study limitations

There are two main limitations to the present study. First, ALP is an essential component of matrix vesicles where it increases for the growth of hydroxyapatite crystal [39]. Furthermore, tissue-nonspecific ALP is distributed in arteries, and is considered to be involved in vascular calcification [39]. We therefore used ALP as surrogate marker of vascular calcification. However, we only examined ALP mRNA levels in vitro and in vivo and ALP activity in vitro, but did not evaluate calcium deposition in vitro and vascular calcification in vivo. It has been shown that calcification does not occur in aortas of STZ-induced type I diabetic rats [40], as is the case with in our experiments. In addition, cultivation in high glucose-containing media does not show calcium deposition in vitro [27]. Therefore, further studies using other animal models of vascular calcification are necessary to evaluate the roles of the Rho–ROCK signaling pathway and the BMPER-mediated signaling pathway in vascular calcification under diabetic conditions.

Another limitation is that in clinical studies whether statins can show an inhibitory effect on vascular calcification is controversial. Kovarnik et al. assessed atherosclerotic plaques using virtual histology intravascular ultrasonography and showed that statins changed the plaques from fibrous and fibro-fatty plaques to necrotic plaques with calcification, suggesting that statins might enhance the density of calcification as part of a healing process [41]. The reason why statins did not show efficacy might be due to their lack of inhibitory effect on progressive calcification, generally detected in clinical imaging, and therefore the starting point to treat might be too late. In the present study, rosuvastatin and BMPER knockdown inhibited ALP activation in HCASMCs, which might reflect a very early stage of vascular calcification.

## Conclusions

Targeting BMPER or BMPER-regulated signaling pathways in vascular smooth muscle cells might be a novel therapeutic target for prevention of vascular calcification.

## Abbreviations

ALP: alkaline phosphatase
BMP: bone morphogenetic protein
BMPER: bone morphogenetic protein-binding endothelial cell precursor-derived regulator
FPP: farnesyl pyrophosphate
GAPDH: glyceraldehyde-3-phosphate dehydrogenase
GGPP: geranylgeranyl pyrophosphate
HCASMC: human coronary artery smooth muscle cell
HMG-CoA: hydroxymethylglutaryl-coenzyme A
HUVEC: human umbilical vein endothelial cell
IPP: isopentenyl pyrophosphate
MYPT1: myosin phosphatase target subunit 1
PCR: polymerase chain reaction
ROCK: Rho-associated protein kinase
RSV: rosuvastatin
SEM: standard error of the mean
siRNA: small interfering RNA
siB: BMPER siRNA
siC: control siRNA
STZ: streptozotocin
TGF: transforming growth factor
